# Megadose Methylprednisolone (MDMP) for Hemangiomatosis

**DOI:** 10.5505/tjh.2012.55706

**Published:** 2012-12-05

**Authors:** Şinasi Özsoylu

**Affiliations:** 1 Retired Prof of Pediatrics, Hematology, Hepatology, Ankara, Turkey; 2 Honorary Fellow of American Academy of Pediatrics; 3 Honorary Member of American Pediatric Society

**To the Editor, **

Dr. Küpeli and his colleagues[1] should be congratulated for their success in treating hemangiomatosis, which could be life threatining depending on the localization of hemangiomas, with propranolol. I prefer the term infantile hemangiomatosis instead of segmental hemangiomatosis[2] for the authors’ patient. My primary reason in writing this letter is to clarify MDMP treatment, which caused severe cushingoid appearance in the patient prior to propranolol treatment. 

In MDMP treatment the timing and duration of MP administration is extremely important; the daily dose must be administered at 0600, either orally or intravenously over the course of 10-15 min. To overcome the bitter taste of MP associated with oral administration, I recommend covering MP with honey. I have used MDMP at 30-100 mg/kg/d as a starting dose for different hematological and non-hematological conditions[3,4] that were resistant or refractory to conventional corticosteroid treatment (1-2 mg/kg/d in divided doses) for several months-years,[3] including hemangiomas, infantile hemangiomatosis, and Kasabach-Merritt syndrome,[5] without observing severe cushingoid appearance. In addition, among 700 patients I observed no or only mild adverse effects of corticosteroids, as reported by others[6]. I have advocated ACTH-corticosteroid homeostasis during MDMP treatment, which differs from pulse MP (in which 1 g of MP is intravenously infused over the course of 3-4 h at any time of the d) and conventional corticosteroid administration (in divided doses). 

MDMP is a type of corticosteroid treatment characterized by ACTH-corticosteroid homeostasis via administering the total daily dose (which starts at 30-100 mg/ kg/d) and gradual tapering[7]. With the exception of acute ITP, it is administered weeks, months, and years without marked adverse effects of steroids. I would like to question the timing and duration of MP administration in the authors’ patient. MDMP was administered over the course of months in a patient with Kasabach-Merritt syndrome, with some increased doses in between which sternum was also involved[5]. MDMP and conventional corticosteroid treatment were comparatively studied by Uysal et al. for the treatment of hemangiomas, and it was observed that MDMP was superior[8]. Lastly, I would like to indicate that I used propranolol 2-5 mg/kg for the treatment of portal hypertension in children [9].

## References

[1] Ku?peli S, Cimen D, Ku?peli BY (2012). Successful treatment with propronolol in a patient with a segmental hemangioma: A case report. Turk J Hematol.

[2] Ozsoylu S (1992). Mega dose methylprednisolone for diffuse infantile haemangiomatosis. Eur J Pediatr.

[3] Ozsoylu S (1990). High-dose methylprednisolone (HIVMP) in hematologic disorders. Hematology Reviews.

[4] Ozsoylu S (2010). Megadose methylprednisolone for hematologic and non hematologic disorders. Turk J Hematol.

[5] Ozsoylu S, Irken G, Gu?rgey A (1989). High-dose intravenous methylprednisolone for Kasabach-Merritt syndrome. Eur J Pediatr.

[6] Bernini C, Carillo M, Buchanon GR (1995). High-dose intravenous methylprednisolone therapy for patients with Diamond- Blackfan anemia refractory to conventional doses of prednisolone. J Pediatr.

[7] Ozsoylu S (2007). How should corticosteroid be used?. Turk Med Sci.

[8] Uysal KM, Olgun N, Erbay A, Sarialioglu F (2001). High dose oral methylprednisolone therapy in childhood hemangiomas. Pediatr Hematol Oncol.

[9] Ozsoylu S, Kocak N, Yu?ce A (1985). Propranolol treatment for portal hypertension in children.. J Pediatr.

